# Spatial distribution of the trace elements zinc, strontium and lead in human bone tissue^☆^

**DOI:** 10.1016/j.bone.2013.07.038

**Published:** 2013-11

**Authors:** B. Pemmer, A. Roschger, A. Wastl, J.G. Hofstaetter, P. Wobrauschek, R. Simon, H.W. Thaler, P. Roschger, K. Klaushofer, C. Streli

**Affiliations:** a Atominstitut, Technische Universitaet Wien, Stadionallee 2, 1020 Vienna, Austria; b Ludwig Boltzmann Institute of Osteology at the Hanusch Hospital of WGKK and AUVA Trauma Centre Meidling, 1st Medical Department, Hanusch Hospital, Vienna, Austria; c 2nd Department, Orthopaedic Hospital Vienna-Speising, Austria; d Karlsruhe Institute of Technology, Institute for Synchrotron Radiation, Hermann-von-Helmholtz-Platz 1, D-76344 Eggenstein-Leopoldshafen, Germany; e AUVA Trauma Centre Meidling, Austria

**Keywords:** Trace elements, Spatial distribution, Human bone, Synchrotron radiation micro X-ray fluorescence analysis, Quantitative backscattered electron imaging

## Abstract

Trace elements are chemical elements in minute quantities, which are known to accumulate in the bone. Cortical and trabecular bones consist of bone structural units (BSUs) such as osteons and bone packets of different mineral content and are separated by cement lines. Previous studies investigating trace elements in bone lacked resolution and therefore very little is known about the local concentration of zinc (Zn), strontium (Sr) and lead (Pb) in BSUs of human bone. We used synchrotron radiation induced micro X-ray fluorescence analysis (SR μ-XRF) in combination with quantitative backscattered electron imaging (qBEI) to determine the distribution and accumulation of Zn, Sr, and Pb in human bone tissue.

Fourteen human bone samples (10 femoral necks and 4 femoral heads) from individuals with osteoporotic femoral neck fractures as well as from healthy individuals were analyzed. Fluorescence intensity maps were matched with BE images and correlated with calcium (Ca) content. We found that Zn and Pb had significantly increased levels in the cement lines of all samples compared to the surrounding mineralized bone matrix. Pb and Sr levels were found to be correlated with the degree of mineralization. Interestingly, Zn intensities had no correlation with Ca levels. We have shown for the first time that there is a differential accumulation of the trace elements Zn, Pb and Sr in BSUs of human bone indicating different mechanisms of accumulation.

## Highlights

• The distribution of zinc, lead and strontium in the microstructure of human bone was analyzed for the first time.
• Synchrotron induced micro X-ray fluorescence analysis was used in combination quantitative backscattered electron imaging.
• Zn and Pb had significantly increased levels in the cement lines compared to bone matrix.
• Pb and Sr levels were correlated with the degree of mineralization.
• Zn intensities had no correlation with Ca levels.

## Introduction

When tissue of living organisms is analyzed by highly sensitive chemical analytic methods, specific chemical elements in very minute quantities (< ppm) can be found. These so called trace elements can be essential and/or non-essential for the living organism [1]. However, the role of many trace elements in tissues e.g. bone is poorly understood [2]. Great efforts have been undertaken to determine the incorporated amounts of various trace elements in bone [3,4]. Since in general the chemical analysis is based on destructive methods, the information about the spatial distribution of the trace elements within the tissue is usually lost. Previous studies lacked spatial distribution and merely differentiated between cortical and trabecular bone [5–10]. New developments in synchrotron radiation technology allow now analyzing in a non-destructive way, spatially resolved trace elements like zinc (Zn), strontium (Sr) and lead (Pb) in bone tissue. For example using synchrotron radiation induced confocal micro X-ray fluorescence analysis (SR μ-XRF) we found a highly specific accumulation of Pb and Zn in the transition zone between mineralized and nonmineralized articular cartilage compared to subchondral bone [11,12]. Moreover this method is also able to detect and map different elements simultaneously [13].

Zn, Sr and Pb are trace elements, present in sufficient concentrations in bone so they can be easily mapped with the multi-elemental SR μ-XRF method. Zn is an important essential trace element in multiple biological processes and a reduced intake may lead to chronic diseases [14]. Zn is also present in bone tissue and it has been reported to play an important role in bone metabolism [15–17]. Studies on the Zn levels in different tissues revealed that most of it is present in bone and for this reason Zn may be considered as an essential component of the calcified matrix [18,19]. Sr is likely a non-essential trace element, but in recent years, studies have shown that Sr is able to influence bone turnover [20] and has been applied in the form of strontium ranelate in therapeutic treatment of osteoporosis. Sr is chemically very similar to calcium (Ca), and can replace Ca, but still little is known about the role of Sr in normal bone metabolism as well as in bone disorders. Pb is a non-essential trace element and represents a highly toxic heavy metal. One of the main threats to human health from heavy metals is associated with exposure to Pb. Exposure to Pb is associated with chronic diseases in the nervous, hematopoietic, skeletal, renal and endocrine systems [21,22]. Pb has been stated also as a potential risk factor for osteoporosis [23] and osteoarthritis [24]. Approximately 95% of the total body Pb burden is stored in skeleton [25] indicating that the bone tissue has a high capacity to accumulate and store Pb. In this context the bone tissue seems to have also the function to keep down the serum levels of such highly toxic elements.

Human bone is essentially composed of a non-homogeneous and non-isotropic arrangement of mineralized collagen fibrils. Cortical and trabecular bones are formed by individual osteons and bone packets (so called bone structural units — BSUs). They are produced at different moments during the (re)modeling cycle by the coordinated activity of bone cells, whereby the osteoblasts synthesize, secrete and deposit the collagenous matrix, which then gradually mineralizes. Thus, each BSU has a certain mineral content depending on the time of deposition [26]. In general these BSUs are connected by a thin layer of mineralized non-collagenous proteins, the so called cement line/layer produced during the remodeling cycle [27]. Only very little data are available regarding the detailed spatial distribution of trace elements within such a bone tissue.

Thus, the aims of this study were to map the trace elements Zn, Sr and Pb in bone tissue and to elucidate the following questions: i) is there a differential accumulation pattern of Zn, Sr and Pb depending on Ca content of mineralized bone matrix in the bone packets, osteons, and interstitial bone? and ii) is the accumulation of Zn, Sr and Pb in cement lines different from that of mineralized bone matrix? Taking into account that the spot size of the confocal SR μ-XRF setup is about 5 times wider than the width of the cement lines the measured intensities are actually a huge underestimate of the real levels of trace elements in this region.

For this purpose we analyzed trabecular and cortical bones from human femoral necks and heads using SR μ-XRF in combination with quantitative backscattered electron imaging (qBEI). qBEI, a well established and validated method [28], was used to visualize the mineralized tissue with a spatial resolution of 1 μm per pixel, to quantify the local bone mineral/Ca content and select the regions of interest for SR μ-XRF measurements in the bone tissue.

## Materials and methods

### Bone samples

For this study bone samples from 14 postmenopausal women have been analyzed: a) Femoral neck samples (n = 10) which had been part of a former study [29,30] and were kindly provided by N. Loveridge (Department of Medicine, University of Cambridge, Cambridge). Five of these samples were from patients suffering from an osteoporotic femoral neck fracture and 5 samples were from forensic autopsies of individuals without metabolic bone diseases age matched with that of osteoporotic fractures. The average age of these individuals was 81.5 years ranging from 74 to 92 years. b) Femoral head samples (n = 4), which were obtained during hip replacement surgery. The individuals suffered an osteoporotic femoral neck fracture and were 60 to 80 years old with an average age of 77.5 years. Measurements were performed in both trabecular and cortical bone regions for the femoral neck samples and only in the trabecular region for the femoral head samples resulting in a total of 35 areas of about 500 μm × 650 μm. The term mineralized bone matrix will describe both the osteons and the interstitial bone in the osteonal bone region and bone packets in cancellous bone region. To the best of our knowledge, none of the patients has been exposed to higher Pb concentrations than the natural levels in their living areas. The study was in accordance with and approved by the local ethics committee (Institutional Review Board of the Medical University of Vienna).

### Sample preparation

As already described in earlier publications [31,32], the samples have been prepared as blocks of undecalcified in polymethylmethacrylate (PMMA) embedded bone tissue. The femoral neck samples were cut in the transversal plane and the femoral head samples perpendicular to the articular surface (frontal plane). The section surfaces were manufactured by grinding with sand paper and subsequently polishing with diamond suspension (3 and 1 μm grain size) on a precision polishing device (PM5: Logitech Ltd., Glasgow, UK) or by milling with a diamond ultra miller (SP2600: Leica Microsystems GmbH, Wetzlar, Germany). The entire embedding and surface preparation procedure was tested to be free of detectable Zn, Sr and Pb contaminations.

### qBEI

Quantitative backscattered electron imaging (qBEI) is a validated technique to visualize and quantify the calcium (Ca) concentration distribution in bone based on the backscattering of electrons from the sample surface in a scanning electron microscope (SEM). Areas with bright gray levels reflect matrix with high Ca content, whereas areas with dark gray levels indicate low Ca content. Cement lines, the transition zones between different bone packets and osteons usually show a higher mineral content than the adjacent mineralized bone matrix [26,33]. More details on the qBEI method can be found elsewhere [31,34].

A SEM (DSM 962, Zeiss, Oberkochen, Germany) was employed to acquire qBEI images using 20 keV electrons leading to an information depth of about 1.5 μm [35]. Images at different magnifications 12-fold for overviews and 200-fold (pixel resolution of about 1 × 1 μm^2^) were obtained to select and define the region of interest (ROI) in bone for SR-μ-XRF analysis similar to a study done previously [32]. Especially areas (bone packets, osteons) containing mineralized bone matrix with different degrees of mineralization have been selected.

### SR-μ-XRF

The properties of synchrotron radiation (SR) including high photon flux, natural collimation, polarization and the possibility to select the energy of the primary photons enabled sensitivities up to the femtogram range and a high spatial resolution in the micrometer range. In previous studies, the combination of a confocal geometry and SR allowed the analysis of trace elements in bone and articular cartilage at the micrometer range with high-sensitivity and high spatial distribution [11,36,37]. Further details on confocal SR-μ-XRF can be found elsewhere [38–42].

**Fig. 1:**
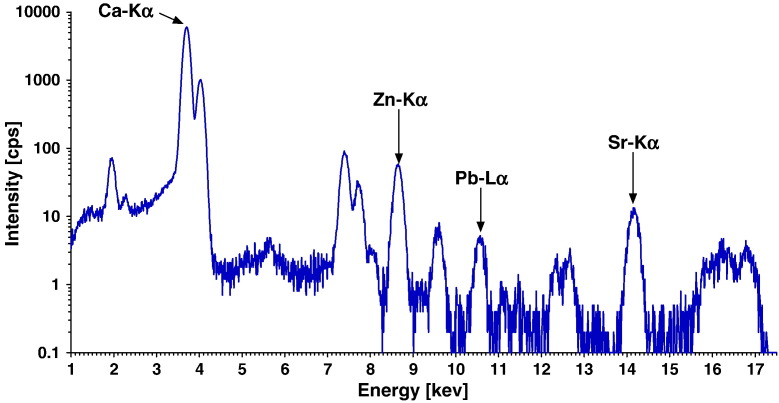
SR μ-XRF spectrum of a measurement point (pixel area) at a cement line. Acquisition time: 10 s; Fluorescence intensity: counts per second (cps) normalized to 100 mA ring current; Peaks not labeled: X-ray Kβ-lines, the scatter peaks (incoherent and coherent) and artificial peaks facts (sum and escape peaks).

The present measurements have been carried out at the FLUO beamline of the ANKA synchrotron facility at the Karlsruhe Institute of Technology Campus North [40,41] applying the same confocal setup as already described previously [32]. The actual excitation energy was 17 keV and the beam size was 17 μm × 12 μm (horizontal × vertical) with a depth resolution of 19 μm at 9.71 keV (Au-Lα). Area scans in the sample surface were performed in the range of 500 μm × 500 μm up to 500 μm × 650 μm with a step size of 15 μm horizontal and 10 μm vertical. Acquisition times longer than 12 s per pixel were found not to show any improvements in the signal to noise ratio of the obtained elemental maps. Especially, the low levels of Pb content required this relatively long acquisition time. The acquired spectra, an example of which is shown in Fig. 1, were processed according to the protocol described in [32].

### Data evaluation

The information about bone tissue structure and mineral content as obtained by qBEI was combined and correlated with the X-ray intensities of the corresponding elemental maps. The 2D data evaluation software ImageJ (v1.44, National Institutes of Health, USA) [43] and custom made routines were applied to pre-process the obtained data prior to statistical evaluation with GraphPad Prism (v4.0c, GraphPad Software, Inc., USA).

#### Regions of interest

**Fig. 2:**
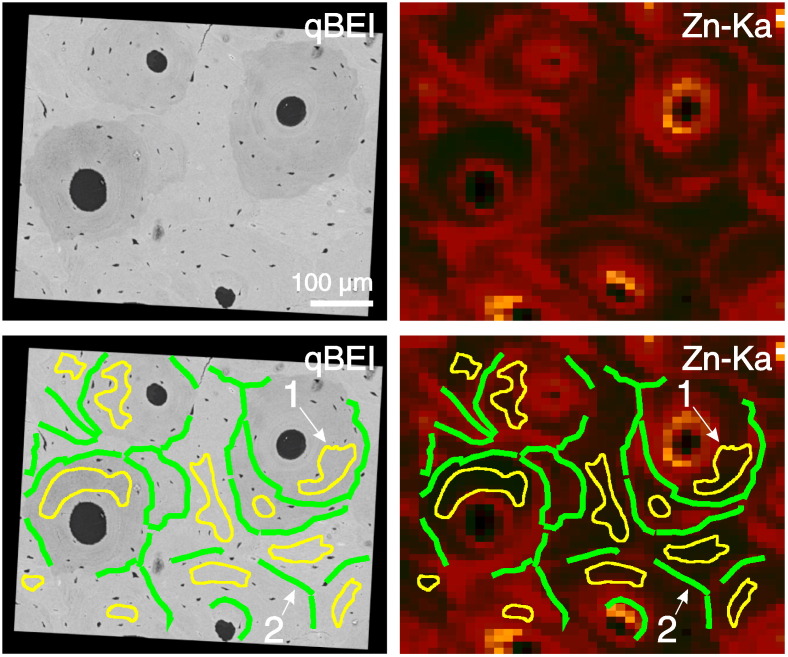
Method to define region of interest (ROI) for SR μ-XRF maps evaluations: 1) Selection of a qBEI image e.g. of osteonal bone region (top-left) together with the corresponding SR μ-XRF map of Zn-Kα (top-right). 2) Identification of homogeneous mineralized bone matrix, cement lines in the qBEI image and outlining them (bottom left): ROI #1 mineralized bone matrix (yellow outlined areas) and ROI #2 cement lines (green lines 10 μm thick). 3) Transfer of the in the qBEI image marked ROIs to the SR-μ-XRF map (bottom-right).

First the qBEI images of high spatial resolution (1 μm per pixel) have been aligned with the corresponding SR μ-XRF maps. Secondly, the ROIs representing mineralized bone matrix and cement lines were indicated in the qBEI images. ROIs of mineralized bone matrix were marked within single structural units (osteon, bone packet) taking care that at least a distance of a few microns (5 to 10 μm) to cracks, cement lines, osteocyte lacunae, haversian canals or trabecular surface was kept. The cement lines themselves were labeled by 10 μm thick lines corresponding the X-ray beam diameter. Finally, these marks/masks in the qBEI image were transferred/overlaid directly to the elemental maps (Fig. 2).

#### Normalization of SR μ-XRF-maps

A general normalization of the XRF count rates for acquisition time and synchrotron-ring current of 100 mA was performed. The XRF intensities of Pb, Zn, and Sr were further corrected for variations in XRF intensities caused by slight changes in the measurement setup between different maps, samples and synchrotron sessions, so that the Pb, Zn, and Sr XRF-intensities between all the maps can be directly compared and treated as measures of elemental content. For this purpose an average factor K (see formula (1)) was evaluated for each map, expressing the mean ratio between Ca as measured by qBEI (wt.% Ca) and Ca as measured by SR μ-XRF(cpsCa). Thus, the multiplication of the SR μ-XRF cps values of Pb, Zn, and Sr from the individual maps with the corresponding K factors leads to a correction/normalization of all the maps based on the absolute Ca values as obtained by qBEI method.

(1) $$K=\frac{1}{n}\sum_{i=1}^{n}\frac{\mathrm{wt}{.\%\mathrm{Ca}}_{i}}{{\mathrm{cpsCa}}_{i}}$$

Formula 1: K = mean normalization factor of one SR μ-XRF map, wt.%Ca_i_ = averaged Ca concentration of mineralized bone matrix ROI_i_ measured by qBEI, cpsCa_i_ = mean Ca-Kα fluorescence intensity of mineralized bone matrix ROI_i_, n = number of the mineralized bone matrix ROIs of the respective map.

#### Statistical evaluation

For each sample the medians of the normalized count rates of Ca, Zn, Pb and Sr for the mineralized bone matrix and the cement line ROIs were calculated. The levels of significance of the differences between mineralized bone matrix and cement lines were tested with the non-parametric Mann–Whitney test for each sample separately. For this purpose all evaluated mineralized bone matrix and cement line ROIs of the respective sample were used. The number of mineralized bone matrix and cement line ROIs was different for all samples. The number of cement line ROIs was larger for all samples. To evaluate the changes in count rate ratios between cement lines and mineralized bone matrix the Wilcoxon signed rank test with the hypothetical median value 1 (= equal elemental distribution) was used.

The significance of the correlation between Ca content and trace element levels of all evaluated mineralized bone matrix ROIs of all samples (n = 402) was tested with the non-parametric Spearman's test. Differences or correlations with p < 0.05 were considered significant.

## Results

It has to be emphasized that the spot size of the confocal SR μ-XRF setup is about 5 times wider than the width of the cement lines. Thus the levels of trace elements in the cement lines presented in the following are actually a huge underestimate of the real levels of trace elements (see details in “Limitations” section).

### Maps of Zn, Pb and Sr in bone tissue

**Fig. 3:**
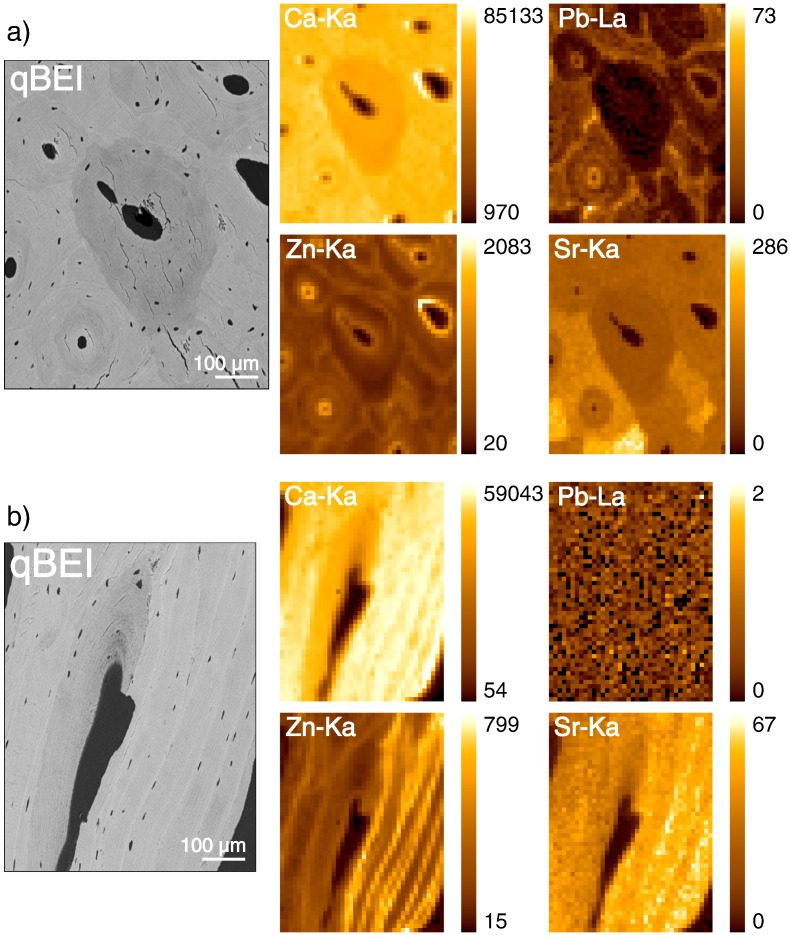
Examples of qBEI images with corresponding SR μ-XRF element maps of Ca, Zn, Pb, Sr: (a) osteonal bone region of the human femoral neck (b) cancellous bone region of the human femoral neck. The color-coded X-ray intensities are normalized to counts per second (cps), 100 mA Ring current and are scaled from minimum to maximum within each individual map. qBEI images show younger bone packets (less mineralized) as darker, and older bone packets (more mineralized) as brighter gray levels. Sample (b) exhibiting multiple parallel cement lines.

In Fig. 3 examples of spatial distribution/maps of the elements Ca, Zn, Pb and Sr in bone tissue are demonstrated: i) the corresponding qBEI images, a) osteonal and b) trabecular bone display regions with different mineral content (dark gray, low and bright gray, high mineral content). ii) None of the elemental XRF maps show a homogeneous distribution within the bone tissue. iii) Zn exhibits a remarkable increase in the cement lines and at the borders to the haversian channels (this region was not evaluated). Zn intensities appear to be rather constant in the mineralized bone matrix. This accumulation of Zn in the cement lines is shown in Fig. 3b. The numerous parallel cement lines seen in the qBEI image correspond with bands of high Zn-Kα intensities in μ-XRF map. iv) Pb also accumulates in the cement lines and in the borders to the haversian channels (this region was not evaluated). Moreover Pb shows a strong correlation to the Ca-content in the mineralized bone matrix. Thus, the central young osteon with low mineralization and therefore low Ca content has a very low Pb content that even the detection limit of the SR-μ-XRF method is reached. In Fig. 3b the Pb levels of the bone samples are so low that the Pb maps exhibit only a noise signal. v) The behavior of Sr distribution is different from Zn and Pb. There is no accumulation at cement lines and haversian channel borders. However there are distinctly visible differences between the mineralized bone matrix of the various osteons.

### Comparison of Zn, Pb and Sr levels between mineralized bone matrix and cement lines

**Fig. 4:**
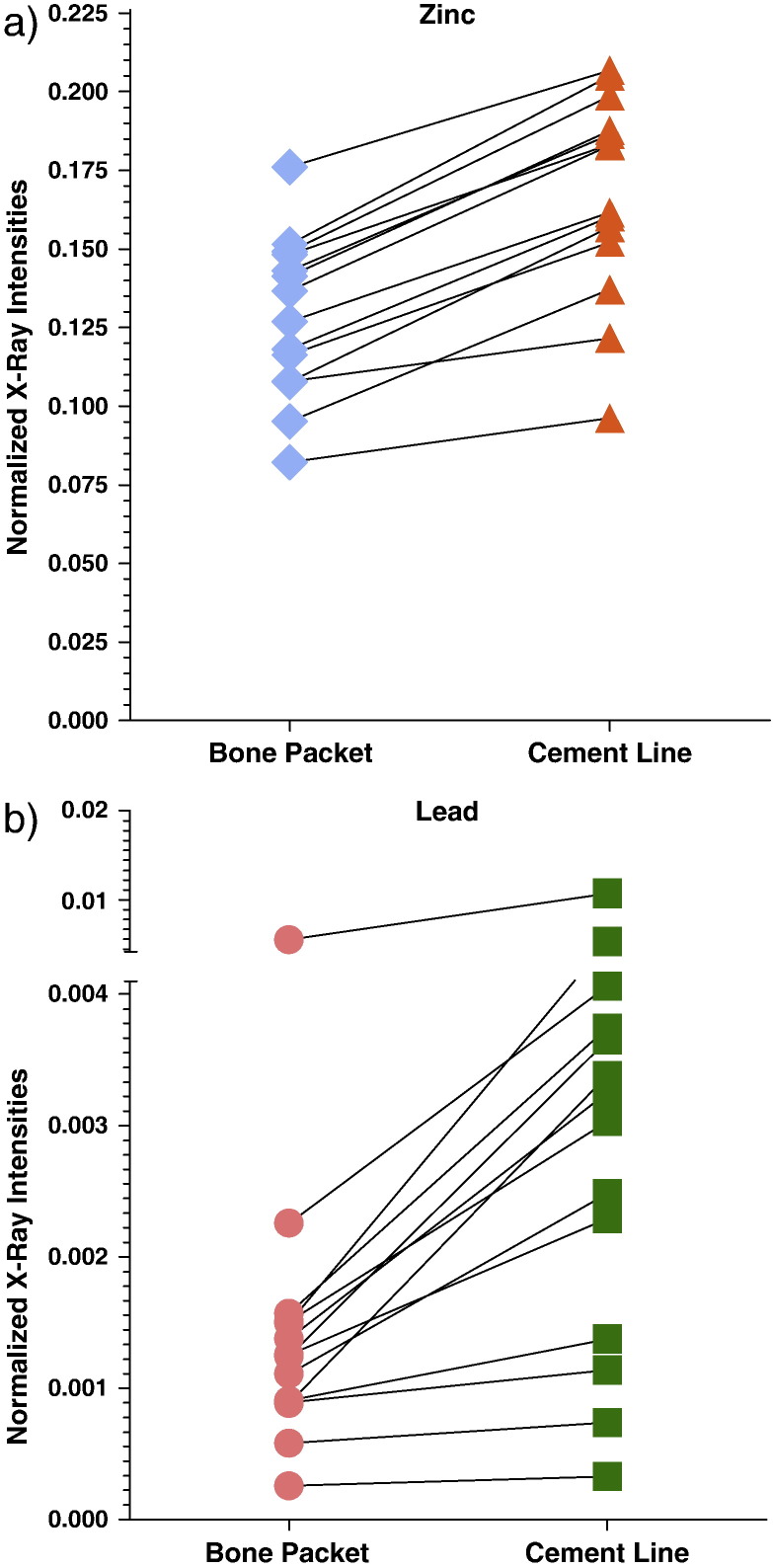
Differential accumulation of Zn and Pb in mineralized bone matrix and cement lines in femoral head and neck samples in the osteonal as well as in the cancellous bone region. For each sample medians resulting from all evaluated mineralized bone matrix and cement lines ROIs of all recorded maps are indicated. Each pair is representing one bone sample.

In all investigated samples we found significantly higher Zn and Pb intensities in the cement lines compared to the mineralized bone matrix (Fig. 4) (p < 0.05 for each individual sample). Even in the sample, which had the lowest Pb level (shown in Fig. 3b), a significantly higher Pb content in the cement lines could be found. There was a large interindividual variation in Zn and Pb XRF intensities of mineralized bone matrix and cement lines (Fig. 4).

**Fig. 5:**
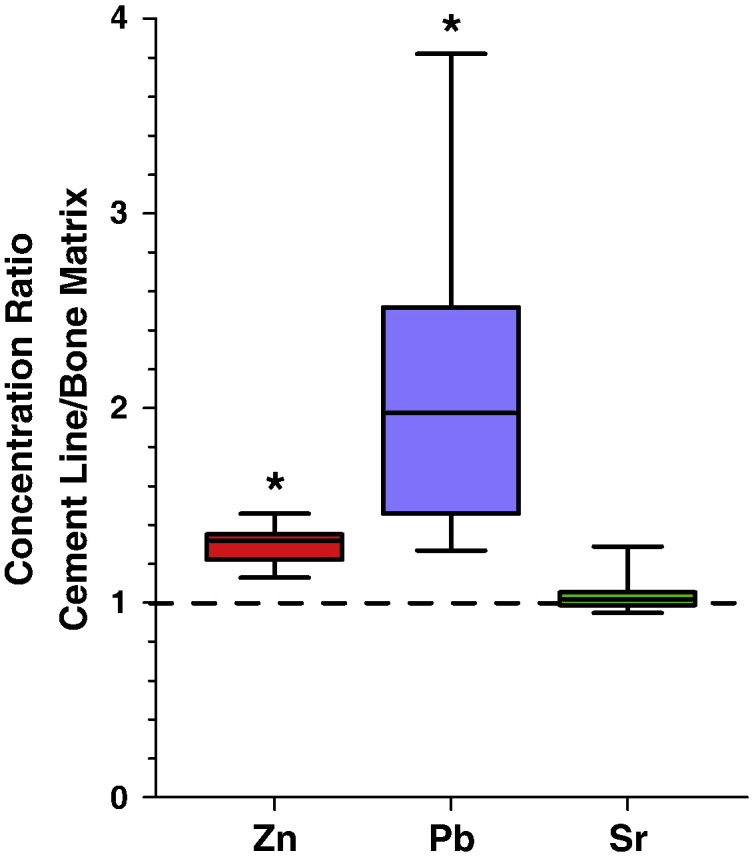
Whisker plots showing median, interquartile range (box) and range (error bars) of concentration ratios of Zn, Pb and Sr between cement lines and mineralized bone matrix. An “*” indicates a significant difference (p < 0.05) from a hypothetical equal distribution (dashed line; Wilcoxon signed rank test).

When analyzing the cement line to mineralized bone matrix ratios for Zn and Pb (Fig. 5) of all samples we found the following: i) Zn content was in median 1.3 times higher (lower quartile: 1.2; upper quartile: 1.4; p < 0.05) in cement line than in mineralized bone matrix; ii) Pb levels were in median 2.0 times higher (lower quartile: 1.5; upper quartile: 2.5; p < 0.05) in the cement line than in mineralized bone matrix; in one sample Pb was 3.8 times increased compared to the mineralized bone matrix (Fig. 5). Thus, we found greater interindividual differences for Pb than for Zn.

In contrast, Sr intensities were not significantly changed between mineralized bone matrix and cement lines.

### Relationship of the mineralization on Zn, Pb and Sr levels in mineralized bone matrix

**Fig. 6:**
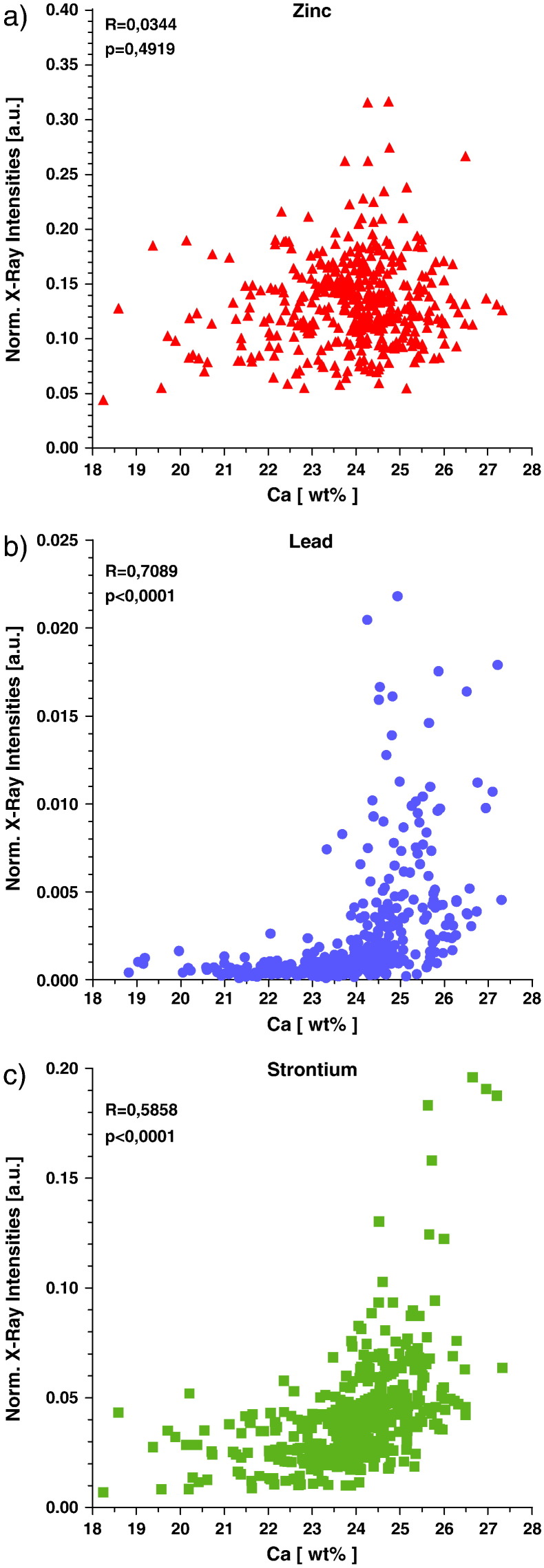
Correlations between Ca content and levels of the trace elements Zn, Pb and Sr in the mineralized bone matrix of the femoral neck and the femoral head samples. X-axis: Ca wt.% as obtained by qBEI. Y-axis: Normalized X-ray intensities using formula (1). The displayed values/data points correspond to all mineralized bone matrix ROIs obtained from all analyzed maps. One data point represents one mineralized bone matrix ROI. Spearman's rank correlation test: R — correlation coefficient, p-value significant for p < 0.05, number of pairs: n = 402.

The correlation of Ca content and trace element levels was evaluated using data obtained from all mineralized bone matrix ROIs (yellow labeled regions in Fig. 2) of all samples. Diagrams showing the relationships of Zn, Pb and Sr to the Ca content are presented in Fig. 6. No correlations between Zn and Ca levels were found, while Pb and Sr showed a non-linear increase with the degree of mineralization, which was significant (p < 0.001; Spearman's rank correlation test).

### Comparison between fractured and non-fractured femoral necks

The analysis of the data from the two subgroups, femoral neck bone with an osteoporotic neck fracture and age matched without fracture, revealed no significant differences in the trace element content and distribution pattern.

## Discussion

Synchrotron radiation induced confocal micro X-ray fluorescence analysis (SR μ-XRF) together with quantitative backscattered electron imaging (qBEI) have been used for the first time to evaluate the spatial distribution of the trace elements Zn, Sr and Pb in bone tissue. The analysis revealed a higher level of Zn and Pb in the cement lines compared to the adjacent mineralized bone matrix. In the bone packets/osteons levels of Pb and Sr were significantly dependent on their Ca content. In contrast, this was not found for Zn.

### Mineralized bone matrix versus cement lines

The cement lines as identified and traced in the qBEI images show consistently higher Zn and Pb values compared to the adjacent mineralized bone matrix indicating a different mechanism of Zn and Pb incorporation/accumulation between these two regions of bone tissue. In contrast to the mineralized bone matrix the cement line (more precise cement surface) is rich with non-collagenous proteins like osteocalcin and osteopontin [27]. During the reversal phase of bone remodeling the cement line is formed, which gets mineralized in general to a higher extent than the adjacent mineralized bone matrix as visualized by backscattered electron imaging. This cement surface layer is exposed to the interstitial fluid until the new bone matrix (osteoid) is deposited by the osteoblasts. During this period Zn and Pb ions present in the interstitial fluid can be accumulated in the deposited cement line material (proteins and mineral) in two ways: a) by uptake of the ions directly in hydroxyapatite and additionally b) by attachment to proteins, which have a high affinity to them. Thus, the increased Pb concentrations in the cement lines may be due to the osteocalcin, which has a higher affinity to Pb than to Ca even at low Pb levels [44,45]. In contrast, Zn is part/cofactor of enzymes like matrix metalloproteinases (MMPs) which are playing an important role in degradation of collagen during the remodeling cycle of bone [46] as well as bone alkaline phosphatase [b-ALP] [47–51]. All synthesized osteoblasts are involved also in the bone matrix mineralization. This increase in Zn levels of the cement line suggests that these enzymes/proteins are stored in the cement lines during the remodeling process. It can be speculated that in a following bone resorption phase the Zn ions are released and again used as cofactor of the enzymes for the subsequent bone formation phase and/or immediately incorporated back into the new formed bone. This is supported by the fact that during bone remodeling Zn is not increasing the serum levels [52–54].

Interestingly, the inter-individual variations of Zn levels are far smaller compared to Pb (Fig. 4a), which suggests that Zn is an inherent component of the cement line rather than dependent of the variations of the period where the cement line is exposed to the interstitial fluid during the remodeling cycle as Pb obviously does. In this context it had to be mentioned that in recent studies regarding the Zn levels observed in the transition zone between mineralized and non-mineralized cartilage (tide mark), a similar differential behavior of Zn and Pb accumulation was found. Zn was distinctly increased without major variations too, while the coincident increase of Pb was higher the longer the tide mark was exposed to the interstitial fluid of the non-mineralized articular cartilage [11,12,36].

In contrast to Zn and Pb, Sr has no accumulation phenomenon in the cement lines that can be observed, though it is well known that Sr^+ 2^ ions are able to substitute Ca^+ 2^ ions. Animal studies suggest that Sr can substitute Ca in almost any physiological process and is almost exclusively deposited in bone [55]. The protein binding affinity of Sr is similar to that of Ca [56]. The dietary amount of Sr can vary widely without occurrence of symptoms of intoxication and it is not under homeostatic control so the blood and serum levels are not kept constant [55]. As it will be elaborated in the limitations below, there might be a coincident increase of Sr with Ca in the cement line, but the relative increase in Ca and Sr is likely too small to be distinguished in a matrix volume of 12 μm (voxel size) with a cement line thickness of only 1 to 2 μm.

### Trace element vs mineralized bone matrix Ca content

Within a BSU the trace elements are uniformly distributed similar to the element Ca. Our hypothesized mechanism of trace element incorporation is therefore, that Zn, Sr and Pb are incorporated into the bone mineral (carbonated calcium hydroxyapatite) during bone formation, when the osteoid gets mineralized by progression of the mineralization front (primary mineralization phase) [26]. The amount of the incorporated trace elements is thereby dependent on the serum levels present. This assumption is strongly supported by the studies we made on Sr incorporation in bone during Sr-ranelate treatments (human and animals [32,57,58]). It could be shown that Sr was incorporated mainly in mineralized bone matrix, which was formed during Sr ranelate treatment. Further, the Sr content was proportional to the Sr serum levels [57]. Moreover, the analysis of the mineral crystal lattice characteristics proved that the Sr ion was incorporated into the apatite crystal lattice [58].

The Pb present in the mineralized bone matrix is most likely accumulated during the mineralization phase similar to Sr. Pb^2 +^ ions in the serum are chemically similar to Ca^2 +^ ions. It has been even demonstrated that Pb^2 +^ is directly competing with Ca^2 +^ at the voltage activated Ca^2 +^ channels [59,60]. Further it has been shown that Pb^2 +^ is able to occupy both Ca^2 +^ sites in the hydroxyapatite (HA) crystal [61–64]. A similar behavior was suggested for Sr^2 +^ ions [55,58]. We assume that as for Sr [57,58,65–68] the amount of Pb incorporated during the mineralization depends on the Pb serum levels. The more Pb^2 +^ ions present in the serum the more Pb ions are incorporated into the bone.

Moreover, in-vitro studies using synthetic HA as well as bovine bone meal found that HA has the ability to accumulate (immobilize) Pb^2 +^, Zn^2 +^, Sr^2 +^ and other divalent metal ions [69–76]. At the moment four different pathways are suggested for the immobilization mechanisms of HA: i) ion exchange process, ii) surface complexation, iii) dissolution and precipitation and co-precipitation [69]. These mechanisms can be expected to be very similar for the other divalent ions. In these studies rather high concentrations of the heavy metals have been used. However according to Bigi et al. [77] and Bückner et al. [78] it is likely that the accumulation mechanisms of HA for Pb^2 +^ are also valid at low concentrations, as they are present in humans. For Pb in bone we have shown that it almost exclusively bonds to carbonated calcium hydroxyapatite [79], which confirms the above assumptions on how Pb is incorporated into the mineralized bone matrix.

Interestingly, despite high intra- and inter-individual variations in Pb (Fig. 4b) and Sr levels, a non-linear increase with Ca-content of the mineralized bone matrix was found (Figs. 6b and c). The over-proportional increase of Pb and Sr at the high mineralization range may be explained by the fact that BSUs with prolonged time of mineralization (secondary mineralization phase) reach a plateau of mineralization (about 26 wt.% Ca) [26]. However, accumulation processes, as already stated above, of Pb^2 +^ and Sr^2 +^ ions in the apatite crystals may be still ongoing with time, after the crystals had stopped growing by ion substitution. Sr^2 +^, Pb^2 +^ and presumably all other divalent metal ions might reach the inner parts of the bone through the vascular system in the haversian channels and bone marrow space, respectively. An animal study using radiostrontium (^85^Sr) showed that the Sr^2 +^ ions pass through the wall of the vascular capillaries by diffusion to reach the interstitial fluids [80]. The same way can be assumed for Pb^2 +^ ions. From the bone marrow space the osteocyte lacunae canaliculi network might be used as pathway for Pb^2 +^ and Sr^2 +^ into the mineralized bone matrix, resulting in the observed overproportional increase of these elements compared to Ca.

Though it has been reported that Zn is concomitantly incorporated with Ca during the mineralization [81], no correlation between Zn and the degree of mineralization like for Sr and Pb was detected by our measurements (Fig. 6a). This is in agreement with prior investigations of Lappalainen et al., who showed that Ca is not a significant factor for explaining the Zn concentrations in bone [82]. Therefore Zn is suggested to be under homeostatic control.

Zn bond to hydroxyapatite (HA) is very likely incorporated during the fast crystallization process as experiments of Tang et al. in 2009 have shown [83]. However the preference coordination site of Zn, the Ca2 site of the HA crystal, would allow the uptake and release of Zn as the Ca2 site framework of the structure is not disrupted [83]. Zn^2 +^ is not simply incorporated by ion exchange processes, but Ca^2 +^ vacancy-defects can act as plausible sites for Zn^2 +^ substitution [84]. As said above, Zn is essential for bone metabolism, as it is part of enzymes important for the remodeling mechanisms of bone and the Zn released during bone remodeling is incorporated back into the bone [46,50,52].

### Limitations

The matching of qBEI images with μ-XRF obtained elemental maps could not be perfectly performed. The different lateral resolutions of SR μ-XRF (~ 10–20 μm) and of qBEI (1–2 μm) make an exact overlay of both maps impossible. Thin features (e.g. cement lines) in the qBEI are blurred in the μ-XRF maps. Furthermore the larger information depth of SR μ-XRF (~ 20 μm for Ca-Kα) compared to qBEI (~ 1 μm) contributes to further blurring. Features close below the surface (e.g. cement lines, or cavities/voids) are not detected by qBEI but might be visible in the corresponding μ-XRF maps. However, superimposing the corresponding SR μ-XRF elemental maps and BE images was found to be very useful in linking bone morphology with X-ray intensities.

An underestimation of Zn and Pb signal intensities in the cement lines is introduced due to the fact that the cement lines are much thinner (in the range of 2 μm) than the focused X-ray beam width. The XRF signal is averaged over a larger matrix volume than the true cement line feature occupies. Hence the obtained data shows a lower limit for the real relative elemental concentration. Assuming a SR μ-XRF voxel size of 12 × 13 × 17 μm^3^ and a cement line of width of 1 μm a 2-fold increase in Pb level in the cement line as measured by μ-XRF might be the result of an actual 34-fold increase.

To determine the signal intensity ratios of Zn and Pb between cement lines and mineralized bone matrix and to further investigate their spatial distribution within the cement line scans or even mappings at nano focus beam lines such as P06 at PETRA III (DESY, Hamburg, Germany) are planned for the future.

No absolute values (wt.%) of Zn, Pb and Sr can be given. Thus, only relative differences between the elements could be reported. Since bone is a complex and highly heterogeneous organic mineral compound, there is no suitable reference material yet for calibration of the experimental setup available, which would have allowed obtaining the absolute concentrations of trace elements corresponding to each measured X-ray count rates.

### Implications

The incorporated Pb, Zn and Sr ions in HA will most likely distort the crystal lattice of the mineral due to the different atomic sizes compared to Ca. This might have negative effects on the stability and strength of the mineral. These effects can probably become relevant at high incorporation levels. However, a 5% replacement of Ca ions by Sr ions occurs in Sr ranelate treatment in postmenopausal osteoporosis [57,58]. The changes in mechanical properties of bone material as measured by nanoindentation could not be observed [57].

The highly toxic effects of Pb on bone cells and bone metabolism and thus bone remodeling are described in detail for high Pb levels of whole body exposure [44,45,60,63,85]. For example, Pb has been shown to alter the Ca homeostasis and perturb the cellular metabolism or activity of osteoclasts [86] and osteoblasts [87–92]. As already stated Pb^2 +^ has a much higher affinity to osteocalcin than Ca^2 +^ [45] and as a consequence Pb^2 +^ influences the binding properties of osteocalcin to the bone minerals negatively [44]. We can speculate that, in principle, the same mechanisms take effect locally, though to a much lower extent, when Pb ions were released in the interstitial fluid during bone remodeling with a normal bone turnover rate. However, the release of Pb stored in the bone can strongly be enhanced in diseases with increased bone turnover.

Medical conditions or diseases, such as osteoporosis, hyperthyroidism, hyperparathyroidism and pregnancy cause an increased bone turnover and are accordingly linked with elevated release of Pb immobilized and stored in the skeleton [22,93,94]. The remobilization of bone Pb back into the circulation is a potentially relevant source of soft-tissue Pb exposure and toxicity long after the external Pb exposure ceased [95]. The Pb in serum may increase to levels which are possibly toxic for inner organs (e.g. the nervous and the hematopoietic system) that are more sensitive to Pb and other heavy metals. Even metabolic processes in the bone are adversely affected by Pb [44,45,60,63,85]. Further Pb has been stated as a potential risk factor for osteoporosis [23], has negative influences on bone healing mechanisms [96] and might affect the articular cartilage tissue [24]. In the present study no significant differences in the trace element content and distribution pattern between bones from individuals with osteoporotic neck fractures and those from age matched healthy individuals without fractures could be detected. However, the sample size was only n = 5.

The main sources of Pb exposure in industrialized countries are derived in the past from leaded water pipes and leaded gasoline. Much effort has been taken to eliminate almost all of these sources [21]. However, the biological half-life of Pb in human bone is about 20 years [97,98]. Thus the bone analyzed from individuals in the age range of 60 to 80 years still had measurable amounts of Pb present. It would be interesting to know how much the environmental Pb uptake is reduced now in young people.

## Conclusions

We have shown for the first time that the distribution of the trace elements Zn, Pb and Sr is not uniform among the structural units of human bone tissues, applying a combination of SR μ-XRF and qBEI. Further cement lines are accumulating Zn and Pb to higher levels than adjacent mineralized bone matrix indicating a possibly different mechanism of Zn, Sr, and Pb uptake. Additionally, it was revealed that in bone structural units the concentration of Pb and Sr depends on the degree of mineralization while this was not the case for Zn.

## Author contributions

All authors were involved in drafting or critically reading the manuscript for important intellectual content, and all authors approved the final version.

Conception and design: B. Pemmer, A. Roschger, A. Wastl, J.G. Hofstaetter, P. Wobrauschek, R. Simon, H.W. Thaler, P. Roschger, K. Klaushofer, C. Streli.

Data acquisition: B. Pemmer, A. Roschger, A. Wastl, R. Simon, C. Streli.

Analysis and interpretation of data: B. Pemmer, A. Roschger, J. G. Hofstaetter, P. Roschger, P. Wobrauschek, C. Streli.

Provision of study material: H.W. Thaler.

Obtaining of funding: C. Streli, P. Roschger.

## Competing interests

None of the authors has any financial or personal relationship with other people or organizations causing conflict of interests.
